# Effects of Environmental Factors on Bird Communities in Different Urbanization Grades: An Empirical Study in Lishui, a Mountainous Area of Eastern China

**DOI:** 10.3390/ani13050882

**Published:** 2023-02-28

**Authors:** Wenwen Zhang, Ying Zhou, Xuelan Fang, Shengjun Zhao, Yi Wu, Haonan Zhang, Liangwei Cui, Peng Cui

**Affiliations:** 1 Key Laboratory for Conserving Wildlife with Small Populations in Yunnan, Southwest Forestry University, Kunming 650224, China; 2 The Provincial Innovation Team of Biodiversity Conservation, Utility of the Three Parallel Rivers Region from Dali University, Dali 671003, China; 3 Research Center for Biodiversity Conservation and Biosafety/State Environmental Protection Key Laboratory on Biosafety, Nanjing Institute of Environmental Sciences, Ministry of Ecology and Environment of China, Nanjing 210042, China

**Keywords:** bird diversity, landscape features, township, urbanization

## Abstract

**Simple Summary:**

In this study, we analyzed the differences in bird diversity in townships with different levels of development to identify the key factors that affect local bird diversity. The results showed that bird diversity increased in moderate urbanization in the mountainous area. At the township scale, bird diversity was determined by the combined effects of landscape diversity and landscape fragmentation. Under the premise of maintaining a high forest coverage rate, increasing landscape diversity and landscape fragmentation is conducive to improving the number, richness, and diversity of bird species. The effect of landscape diversity on bird species numbers, bird richness, and bird diversity is stronger than that of landscape fragmentation. In future urban development planning, heterogeneity and diversity of the landscape pattern can be increased by constructing an urban biological habitat in order to maintain and improve biodiversity. The results obtained in this study provide a practicable reference for formulating biodiversity conservation strategies and constructing reasonable and safe biodiversity patterns.

**Abstract:**

The rapid development of urbanization has changed landscape patterns and biological habitats severely and, therefore, affected biodiversity. In this study, we selected 75 townships in Lishui, a mountainous area in eastern China, to conduct bird surveys for two years. We analyzed the birds’ composition characters in townships with different levels of development in order to identify the effects on bird diversity of the urban development level, land cover pattern, landscape pattern, and other factors. In total, 296 bird species from 18 orders and 67 families were recorded between December 2019 and January 2021. A total of 166 species of birds belonged to *Passeriformes* (56.08%). The seventy-five townships were divided into three grades by K-means cluster analysis. The average number of bird species, richness index, and diversity index were higher in G-H (highest urban development level) compared with the other grades. At the township level, landscape diversity and landscape fragmentation were the key factors that positively affected the bird species number, diversity index, and richness index. Landscape diversity had a greater effect than landscape fragmentation, particularly on the Shannon–Weiner diversity index. The diversity and heterogeneity of urban landscapes could be improved by constructing biological habitats in future urban development planning to maintain and increase biodiversity. The results obtained in this study provide a theoretical basis for urban planning in mountainous areas, and a reference for policymakers to formulate biodiversity conservation strategies, construct reasonable biodiversity patterns, and solve practical biodiversity conservation problems.

## 1. Introduction

The rapid development of cities changes very much of our environment [1]. Urbanization affects biological habitats, including the destruction and loss of habitats, thereby affecting the diversity of plants, insects, and vertebrates, especially birds [2]. Reducing the impact of urban development on biodiversity and reaching a harmonious balance between man and nature is the goal of future urban development, and also a hot topic in biodiversity research [3]. Birds are important components of biodiversity and the most common vertebrates in cities. Bird species are important indicators of the status of urban ecological environments and biodiversity [4]. Given the importance of birds for urban ecological environments and the well-being of residents [5], it is particularly relevant to conduct ornithological research in the context of rapid urbanization.

The present study analyzed the impacts of urban development on bird diversity [6]. The research areas were mostly urban green spaces/parks [7], and locations with different levels of urban development were qualitatively evaluated [8]. From the perspective of the whole urban area, expanding the sampling unit, such as considering a county or township as the smallest unit, can reduce the limitations caused by sampling and more comprehensively reflect the situation for a whole city. The environmental factors that affect bird diversity are considered to vary significantly at different spatial scales [9,10,11]. On a large regional scale, climate, climate stability, and regional areas are among the main factors to affect bird diversity [12,13]. At the local scale, the land-use pattern, landscape pattern, and socio-economic development may be more important for the distribution of biodiversity, especially bird diversity [14]. At even smaller scales, such as urban green spaces and parks, the vegetation structure, green space areas, and habitat characteristics are major factors that influence bird diversity [2,15].

Lishui is located in a mountainous area in a subtropical region of eastern China and was selected as the research site for this study because of its rich bird resources, with 370 species, and most of them are resident bird species (129 species), followed by winter migrants (112 species) [16]. In this study, bird surveys were conducted for 2 years in 75 townships in Lishui. A quantitative method was used to classify the development grade of each township, and the key factors that could affect bird diversity distribution were identified. In particular, we aimed (1) to determine the characteristic bird diversity in townships with different development levels in a mountainous area of eastern China; (2) to identify the key factors that might affect bird diversity at the township level; and (3) to determine how to allocate land use and landscape patterns in a scientific and reasonable manner in order to maintain and increase urban biodiversity under inevitable urban expansion and development. The results obtained in this study may provide a theoretical basis for urban planning and biological habitat design in the future development of cities in mountainous areas. In addition, our findings could provide a reference to allow decision-makers to formulate biodiversity conservation strategies, construct reasonable biodiversity patterns, and solve practical problems in biodiversity conservation.

## 2. Materials and Methods

### 2.1. Study Area

**Figure 1:**
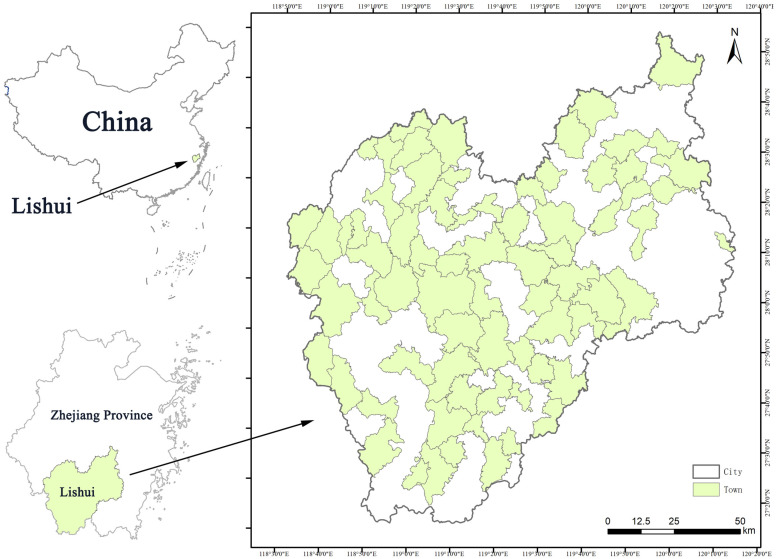
Locations of 75 townships in Lishui. The black line represents the boundary of the Lishui administrative region and the green regions are the 75 townships (drawn by the author).

Lishui City (118°41′–120°26′ E, 27°25′–28°57′ N) in Zhejiang Province is a typical city in a mountainous region of eastern China (Figure 1). Lishui has a total area of 17,275 km^2^ and a population of 2707400 at the end of 2020. Lishui City is a subtropical monsoon climate zone. The average annual temperature is 17.8 °C. Lishui is located in the Wuyi mountain series in a subtropical region, and the forest coverage rate in the city is about 81.7%. In this mountain series, 3573 peaks are greater than 1000 m above sea level. The southwest mainly contains medium-sized mountains and the northeast is dominated by low mountains, with medium-sized mountains and the Bihu Plain, Songgu Plain, and Huzhen Plain in between. The main water systems are the Oujiang River, Qiantang River, and Feiyun River. Thus, Lishui was selected as a representative city for studying the urban bird diversity in a mountainous area [17].

In the last 10 years, rapid urban construction development and urban land-scale expansion have occurred in Lishui. The urbanization rate was 61.8%. Between 2010 and 2020, the urban area increased by 280.2 km^2^, i.e., an increase of 67.5%, mainly due to losses of forest and farmland ecosystems. This rapid development has greatly impacted biodiversity, which may lead to the loss of habitat for some species and a decrease in bird diversity. In this study, bird diversity was investigated in 75 townships (Figure 1) with different development levels, which were identified as research sites according to the township population, population density, township area, urbanization rate, and other indicators.

### 2.2. Bird Surveys

Between December 2019 and January 2021, bird surveys were conducted in the 75 townships in Lishui during each quarter in the spring (April–May), summer (July–August), autumn (October–November), and winter (December–January). Two or three transects were selected in each township. In total, 188 transects were investigated. Surveys were conducted on sunny days during time periods with frequent bird activities (7:00–10:00 and 15:00 to sunset in the spring and autumn; 5:00–8:00 and 17:00 to sunset in the summer; 9:00–11:00 and 15:00 to sunset in the winter). The line transect method was used to conduct the bird surveys. The surveyor walked along the transects at a speed of 1–2 km h^–1^ and recorded the species and number of each species observed or heard within 50 m of the transect’s center. Each transect had a length of 1–1.5 km.

Bird species were identified and classified referring to “*A Checklist of Classification and Distribution of the Birds of China*” (Third Edition) [18]. Conservation status (least concern, near-threatened, vulnerable, endangered, and critically endangered) was based on IUCN [19].

### 2.3. Selection of Environmental Factors and Data Acquisition

Land-use data (2020) from Landsat TM/ETM images with a resolution of 30 m were used as the main data sources. Erdas 9.3 software was used to interpret and quantify the areas of various land-use types. The land-use factors included the following indicators: proportion of forest area (PFOR), proportion of shrub area (PSHR), proportion of grassland area (PGRA), proportion of wetland area (PWET), proportion of bare land area (PBAR), proportion of farmland area (PFAR), and proportion of urban area (PURN) [20].

GIS technology and FRAGSTATS 4.1 software were used to analyze the landscape pattern structure in each township, where the numbers of patches and average patch areas were calculated. The landscape heterogeneity indexes included the landscape diversity index, landscape evenness index, and landscape fragmentation index [21].

The urbanization index included the township populations, population densities, and township area size [22], where the data were derived from the Statistical Yearbook of Lishui in 2021.

### 2.4. Classification of Townships

Quantitative classification of the township development grades allows us to more accurately explore the effects of the urban structure and landscape pattern on the biodiversity distribution [23]. K-means clustering [24] was conducted to divide the 75 townships into different development grades according to factors including the total population, population density, township area, and the proportion of the area comprising non-natural ecosystems (urban and farmland). The K-means clustering results were divided into four grades. The results of 5 clustering indexes of grades 1 and 4 were similar, so, we combined grades 1 and 4. Finally, the seventy-five townships in the study area were divided into three grades, where G-H represented townships with the highest degree of urban development, G-M represented townships with a moderate degree of urban development, and G-L represented townships with a low degree of urban development. The characteristics of the different grades are shown in Table 1.

### 2.5. Data Analysis

#### 2.5.1. Bird Diversity

The number of bird species, richness index, diversity index, and evenness index were used to measure the level of bird diversity in each township [25].

(1) Bird species comprised the total number of bird species observed in four surveys.

(2) The Shannon–Weiner diversity (H_B_) of bird species was calculated using the following formula:

(1) $$H_{B}=-\sum_{i=1}^{S}P_{i}\ln\left(P_{i}\right),$$

where P_i_ is the ratio of the number of individuals from bird species i relative to the total number of individuals in the community and S is the number of bird species.

(3) The richness index (R_B_) comprising the Pielou coefficient was calculated as:

(2) $$R_{B}\;=\left(S_{B}\;–\;1\right)/\mathrm{Ln}\left(N_{B}\right)$$

where S_B_ is the number of bird species and N_B_ is the sum of the number of individuals of all bird species.

(4) The evenness index (J_B_) was calculated as:

J_B_ = H_B_/H_B_max, (3)

where H_B_max is the theoretical maximum diversity index and H_B_max = Ln(S_B_).

#### 2.5.2. Landscape Index 

According to previous research, the landscape heterogeneity indexes included the landscape diversity index, landscape evenness index, and landscape fragmentation index [21].

(1) The Shannon–Weiner landscape diversity (H) was calculated as:

(4) $$H=-\sum_{i=1}^{S}P_{i}\ln\left(P_{i}\right),$$

where S is the total number of landscape types in a township and Pi is the proportion of the landscape area for type i relative to the total area.

(2) The landscape evenness index (J_B_) was calculated as:

J = H/Hmax,(5)

where Hmax is the theoretical maximum diversity index and Hmax = Ln(S).

(3) The landscape fragmentation index was calculated as:

D = (N − 1)/NC,(6)

where N is the total patch number and NC is the average patch area in all landscape types.

#### 2.5.3. Statistical Analyses

One-way analysis of variance was conducted to test for differences in the bird species, richness index, diversity index, and evenness index between townships with different grades [26]. Species accumulation curves were used to assess whether the sample sizes were adequate [27] and to estimate the community richness in towns with different grades by applying the “vegan” package in R 4.1.6.

Variance inflation factors (VIFs) were calculated to assess the collinearity among environmental variables [28]. After removing two variables with strong collinearity comprising the total population and landscape evenness index, the VIF values for all environmental variables were less than 3. Finally, the number of bird species, richness index, diversity index, and evenness index were used as response variables. 

A multiple stepwise regression analysis [28] was conducted using 10 indicators comprising the township population density, township area, shrub area proportion, grassland area proportion, wetland area proportion, bare land area proportion, farmland area proportion, urban area proportion, landscape diversity index, and landscape fragmentation index, as predictor variables. All analyses were performed using the “vegan” package in R 4.1.6 and with IBM SPSS Statistics 25.

## 3. Results

### 3.1. Bird Diversity

Between December 2019 and January 2021, 296 species belonging to 18 orders and 67 families were recorded in the study areas (Table 2). Most bird species belonged to *Passeriformes* (166 species, 56.08%), whereas *Suliformes*, *Bucerotiformes* and *Trogoniformes* had the lowest number of representatives (one species each).

In particular, sixty-one of the bird species are under special protection in China, including seven species under first-class state protection and fifty-four species under second-class state protection. Among the birds under special protection, the top five orders were *Accipitriformes* (eighteen species, 29.5%), *Passeriformes* (ten species, 16.39%), Strigiformes (eight species, 13.11%), *Falconiformes* (five species, 8.20%), and *Galliformes* (five species, 8.20%).

Among the IUCN Red List of Threatened Species, the least concerned species comprised the greatest proportion (84.12%, *n* = 249). Two critically endangered (CR) species were recorded comprising the Siberian crane (*Grus leucogeranus*) in Huzhen town and yellow-breasted (*Emberiza aureola*) bunting in Shitang town and Bihu town. Four endangered (EN) species were recorded comprising the scaly-sided merganser (*Mergus squamatus*), white-eared night heron (*Gorsachius magnificus*), Cabot’s tragopan (*Tragopan caboti*), and black-faced spoonbill (*Platalea minor*). Six vulnerable (VU) species and thirty-five near-threatened (NT) species were also recorded.

In terms of the different residence types, most were resident bird species (136 species, 45.95%), followed by winter passing migrants (58 species, 19.59%), visitors (55 species, 18.58%), and summer visitors (47 species, 15.88%).

### 3.2. Analysis of Bird Diversity in Townships with Different Grades 

**Figure 2:**
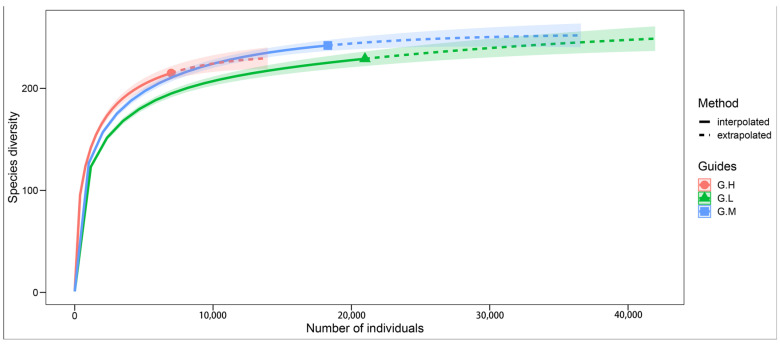
Sample completeness curves for bird communities in three township grades. The filled circles represent the sample coverage. Red represents G-H, blue represents G-M, and green represents G-L.

The species accumulation curves obtained for the three grades were characterized as gradually flattening as the number of individuals increased (Figure 2). More individual birds made small contributions to the new species records, thereby suggesting the gradual saturation of bird species. The sampling integrity of the bird survey in each township was greater than (99%), which indicates that the overall data set was reasonable and suitable for further analysis. The bird species recorded in different township grades differed significantly (*p* < 0.001).

The total number of species was highest in G-M (242 species), followed by G-L (229 species) and G-H (215 species). However, the average number of bird species was highest in each township in G-H (*n* = 7, mean ± standard deviation (SD) = 79.71 ± 18.33), and the average number of bird species decreased as the township development grade decreased (G-M, *n* = 23, mean ± SD = 59.87 ± 19.04; G-L, *n* = 45, mean ± SD = 50.13 ± 13.77).

**Figure 3:**
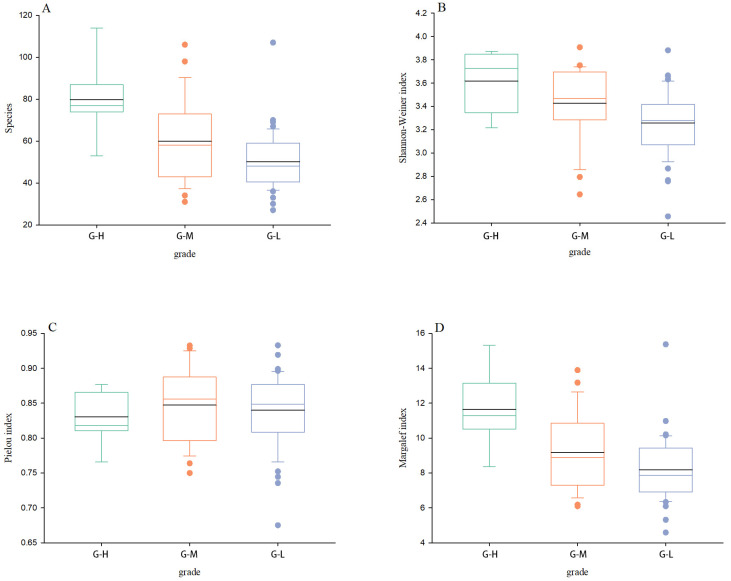
Comparison of average bird species (**A**), Shannon–Weiner index (**B**), Pielou index (**C**), and Margalef index (**D**) in three township grades. In the box plot, the black horizontal line represents the average values, the colored horizontal lines represent the median, dots represent the outliers.

The bird diversity index (*p* = 0.003) and richness index (*p* < 0.001) differed significantly among township grades, but there was no significance. Figure 3 shows that the bird diversity index, evenness index, and richness index were highest in G-H. The bird richness index followed the order of G-H (*n* = 7, mean ± SD = 11.638 ± 2.179) > G-M (*n* = 23, mean ± SD = 9.165 ± 2.114) > G-L (*n* = 45, mean ± SD = 8.183 ± 1.821). The diversity index followed the order of G-H (n = 7, mean ± SD = 3.628 ± 0.255) > G-M (*n* = 23, mean ± SD = 3.427± 0.325) > G-L (*n* = 45, mean ± SD = 3.258 ± 0.271). The evenness index followed the order of G-H (*n* = 7, mean ± SD = 0.831 ± 0.039) > G-M (*n* = 23, mean ± SD = 0.847 ± 0.052) > G-L (*n* = 45, mean ± SD = 0.840± 0.052).

**Figure 4:**
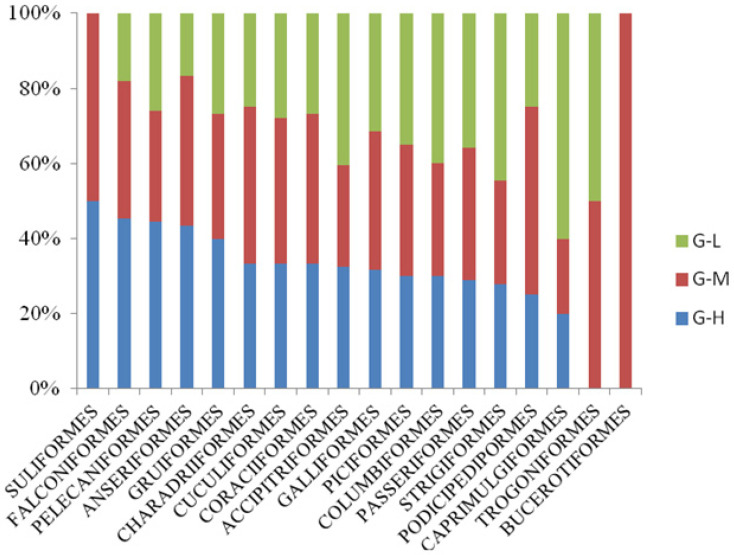
Comparison of bird species from different orders in the three township grades. Green represents G-L, red represents G-M, and blue represents G-H. The lengths of the different colored columns indicate the relative number of bird species from an order in different towns.

In terms of the composition of birds, *Passeriformes* comprised the most abundant order in the three township grades (G-L > G-M > G-H). Figure 4 shows that the waterbird species represented by *Anseriformes*, *Pelecaniformes*, *Gruiformes*, and *Charadriiformes* were more abundant in G-H. Significantly more forest bird species represented by Accipitriformes and *Strigiformes* were found in G-L than in G-H and G-M. In *Trogoniformes*, the red-headed trogon was only recorded in G-M and G-L.

### 3.3. Environmental Variables Affect Bird Diversity

As shown in Table 3, the multiple stepwise regression analysis results obtained two explanatory models for the three response variables comprising bird species, diversity index, and richness index. The model that included the landscape diversity index (H) and landscape fragmentation index (D) was best at explaining the number of bird species, diversity index, and richness index. The models are as follows:

Y_bird specie_ = 30.489+3.118 × H+14.327 × D (7)

Y_diversity index_ = 2.948+0.045 × H+0.240 × D (8)

Y_richness index_ = 6.168+0.354 × H+1.351 × D (9)

Multiple stepwise regression analysis did not obtain a reasonable model for explaining the bird evenness index. The 10 predictor variables tested in this study had no significant effects on the bird evenness index.

## 4. Discussion

### 4.1. Bird Diversity in Different Township Grades 

Negative effects of urbanization on bird diversity have been confirmed in many previous studies [29]. In this study, we determined bird diversity in townships with different grades of development; however, our results showed that the number of bird species, richness index, and diversity index were significantly higher in the townships with the highest level of development. This finding appears to be different from most previous studies. The study location is a mountainous area and the average proportion of the 75 townships covered by natural ecosystem areas is 78.67%. The townships in the study area with the highest level of urban development still had a high proportion of their areas covered by forest ecosystems (mean = 56.63%). Thus, appropriate urban development (mean = 16.12% of urban ecosystem area) is conducive to increasing the diversity of birds. This trend agrees with the moderate disturbance hypothesis, where biodiversity peaks under an intermediate level of disturbance [30,31,32], which can be achieved through urban wetland parks [33], contiguous farmland interspersed with forest land, and other patterns. Our results are consistent with the conclusions obtained by Marzluf et al., who studied areas with 20–90% forest coverage and found that bird diversity peaked under forest coverage of 50–60% [34]. Palomino et al. also showed that the bird species richness was highest in areas with an urban building coverage of 15–28% [15]. In conclusion, moderate urbanization increases the diversity of habitats, and thus the richness and diversity of bird communities.

### 4.2. Key Environmental Factors That Affected Bird Diversity

The factors that affect urban bird diversity have been studied for many years. It is generally considered that habitat patterns such as landscape heterogeneity, landscape fragmentation, and habitat connectivity are the main factors that affect urban bird diversity at the urban scale [35]. In the present study, the multiple stepwise regression models obtained based on the bird diversity index and various factors showed that landscape diversity and landscape fragmentation had significant positive effects on bird diversity, and they were the key variables that affected the number of bird species, diversity index, and richness index.

In terms of the impact of landscape diversity, our findings are consistent with those obtained in most previous studies [2]. It is considered that improving landscape diversity is conducive to increasing bird diversity. Townships with generally high landscape diversity have large areas covered by forest ecosystems, but other ecosystem types are also present, such as farmland, wetlands, and towns. Forests provide adequate food and habitat for most bird species [36], and the increment of other landscape types can enhance bird diversity by providing the habitats available for birds that are more dependent on wetlands, farmland, and towns, thereby providing more ecological niches and refuges [3].

We also found that at the township level, bird diversity was strongly determined by landscape diversity and landscape fragmentation, and the effect of landscape diversity was stronger than landscape fragmentation. Similarly, De Camargo et al. suggested that the response of birds to habitat quantity is significantly stronger than that to landscape fragmentation at the landscape level [37]. However, the positive or negative effect of landscape fragmentation has been debated. Numerous studies have shown that the relationship between landscape fragmentation and bird diversity varies in different threshold ranges [34,37,38]. According to our findings, in the study location where the urban landscape area did not exceed 45%, increased landscape fragmentation improved bird diversity because the large and contiguous forests in this landscape pattern were divided by small farmland areas, towns, and other habitats, which increased the number of patches in the landscape. However, the increased landscape fragmentation also increased landscape diversity, thereby maintaining higher bird diversity and richness [38]. Fahrig et al. also concluded that landscape fragmentation increases species richness by increasing landscape diversity. In addition, Deng et al. argued that bird densities are higher on small forested islands than on large islands [39].

### 4.3. Suggestions for Preserving Biodiversity during Urban Development

Maintaining and even improving urban biodiversity must be considered during inevitable urban development planning. It has been shown that forest agricultural mosaic landscapes can enhance bird diversity in farmland landscapes, and wetland patches and mosaic patterns with surrounding forests can also promote the coexistence of organisms that depend on different habitats [40]. Therefore, in urban planning and management, in addition to maintaining a high forest coverage rate, the heterogeneity and diversity of the landscape pattern should be increased in an appropriate manner to enhance local biodiversity, which can be achieved by designing urban biological habitats. The existing nature reserves, forest parks, wetland parks, and other protected natural areas in cities are all biological habitats with good foundations. In city planning, attention should be paid to the design of various types of habitats to improve the diversity of the landscape [15]. Therefore, it is very important to increase the number of biological habitats comprising grassland, wetland, farmland, and other types in mountainous cities. In addition, special landscape types should be preserved and promoted. For example, the meadow landscape distributed in high-altitude areas in the current study area should be strongly protected to maintain the diversity of birds, such as the russet bush warbler (*Locustella mandelli*) and buff-throated warbler (*Phylloscopus subaffinis*), which are highly dependent on these landscape types.

The two approaches that can be applied to establish biological habitats are ecosystem-centered and species-centered [41], where the former focuses on the overall design from the perspective of the landscape and the latter focuses on the design of biological habitats from the perspective of protecting a certain species or group. Birds have high activity levels and they are dependent on specific habitats. Most birds can select a fixed habitat that provides a suitable environment. The diversity of other groups can be indirectly protected by protecting and promoting certain bird habitats, thereby improving biodiversity. For example, in order to protect the scaly-sided merganser (assessed as EN level in the IUCN Red List), we can protect the river landscape where it lives, preserve the farmland and village landscapes around the river landscape as buffer zones to reduce excessive human disturbance, and treat the scaly-sided merganser as an umbrella species to improve the habitat quality for other birds in *Anseriformes*, which accounted for 5.41% of the birds in the study area [42].

## 5. Conclusions

In this study, we investigated bird diversity and distribution patterns at the township level in Lishui, a mountainous area of eastern China. We obtained the following main results. (1) In the mountainous area, moderate urbanization increased the diversity of habitats, which may harbor an increased number of bird species and richness, and improve the diversity of bird communities. (2) At the township scale, bird diversity was determined by the combined effects of landscape diversity and landscape fragmentation, and the effect of landscape diversity was stronger than that of landscape fragmentation. (3) Under the premise of maintaining a high forest coverage rate, maintaining landscape diversity was conducive to increasing the number of bird species, richness, and diversity. Landscape fragmentation is improved by adding wetlands, grasslands, farmlands, urban parks, etc. In this case, the increase in landscape fragmentation might improve the number of bird species, richness, and diversity. In addition, due to data acquisition limitations, the predictor variables used to determine effects on bird diversity did not include socio-economic development level indexes, but the effects of these factors could be investigated in future research.

Based on our conclusions, we suggest that in future urban planning and management, the heterogeneity and diversity of landscape patterns can be increased by constructing urban biological habitats to maintain and enhance local biodiversity. The results obtained in our study provide a theoretical basis for urban planning in similar mountain cities during future development. In addition, our results provide a reference for decision-makers to formulate better biodiversity conservation in city development plans and practical biodiversity conservation problems.

## Figures and Tables

**Table 1:** Average values (± one standard error) for characteristics of each township grade.

	G-H Number of Towns: 7	G-M Number of Towns: 23	G-L Number of Towns: 45
Population Density	402 ± 186	122 ± 83	93 ± 61
Total Population	56923 ± 18080	18347 ± 5794	8314 ± 3694
Township Size	158.44 ± 61.57	181.98	104.98
PURN	16.12 ± 12.95	3.31 ± 0.94	2.65 ± 2.13
PFAR	27.25 ± 7.30	15.87 ± 7.25	16.35 ± 8.59
PFOR	49.29 ± 14.64	73.98 ± 9.31	75.31 ± 10.10
PSHR	1.89 ± 20.09	1.35 ± 0.90	1.08 ± 0.66
PGRA	2.91 ± 0.72	2.54 ± 2.27	2.85 ± 1.56
PWET	2.22 ± 0.41	2.57 ± 2.91	1.24 ± 1.40
PBAR	0.32 ± 0.15	0.38 ± 0.40	0.52 ± 0.35
Landscape Diversity Index	1.2037 ± 0.1057	0.9896 ± 0.5019	0.7741 ± 0.2066
Landscape Evenness Index	0.6186 ± 0.0543	0.5086 ± 0.2579	0.3978 ± 0.1062
Landscape Fragmentation Index	8.1571 ± 4.1850	4.3361 ± 0.3547	3.3444 ± 1.6081

**Table 2:** Compositions of bird communities observed in 75 townships.

Orders	Families	Proportion of Total Families	Species	Proportion of Total Species
PASSERIFORMES	38	56.72%	166	56.08%
CHARADRIIFORMES	6	8.96%	22	7.43%
ACCIPITRIFORMES	2	2.99%	18	6.08%
ANSERIFORMES	1	1.49%	16	5.41%
PELECANIFORMES	2	2.99%	14	4.73%
GALLIFORMES	1	1.49%	8	2.70%
STRIGIFORMES	1	1.49%	8	2.70%
GRUIFORMES	2	2.99%	7	2.36%
CUCULIFORMES	2	2.99%	7	2.36%
PICIFORMES	1	1.49%	7	2.36%
CORACIIFORMES	3	4.48%	6	2.03%
FALCONIFORMES	1	1.49%	5	1.69%
COLUMBIFORMES	1	1.49%	4	1.35%
CAPRIMULGIFORMES	2	2.99%	3	1.01%
PODICIPEDIPORMES	1	1.49%	2	0.68%
SULIFORMES	1	1.49%	1	0.34%
TROGONIFORMES	1	1.49%	1	0.34%
BUCEROTIFORMES	1	1.49%	1	0.34%
Total	67	100%	296	100%

**Table 3:** Multiple linear regression models based on relationships between environmental variables (predictor variables) and the number of bird species, bird diversity index, and bird richness index (response variables).

Response Variables	Model	Input Variables	R^2^	F	Significance
Bird Species	Model1	landscape fragmentation index	0.214	21.148	*p* < 0.001
	Model 2	landscape diversity index	0.281	15.429	*p* = 0.007
		landscape fragmentation index			
Bird Diversity Index	Model 1	landscape fragmentation index	0.155	14.565	*p* < 0.001
	Model 2	landscape diversity index	0.218	11.317	*p* = 0.011
		landscape fragmentation index			
Bird Richness Index	Model 1	landscape fragmentation index	0.184	17.722	*p* < 0.001
	Model 2	landscape diversity index	0.221	11.474	*p* = 0.039

## Data Availability

The data will be provided upon request to the corresponding author.
